# Investment in online self-evaluation tests: A theoretical approach

**DOI:** 10.1186/1752-2897-2-3

**Published:** 2008-04-15

**Authors:** Francesco de Gara, William T Gallo, Jonathan I Bisson, Jerome Endrass, Stefan Vetter

**Affiliations:** 1 Centre for Disaster and Military Psychiatry, University of Zurich, Zurich, Switzerland; 2 Department of Epidemiology and Public Health, Yale University School of Medicine, New Haven, USA; 3 Department of Psychological Medicine, Cardiff University, Cardiff, UK; 4 Psychiatric/Psychological Service, Criminal Justice System Canton of Zurich, Zurich, Switzerland

## Abstract

**Background:**

Large-scale traumatic events may burden any affected public health system with consequential charges. One major post-disaster, expense factor emerges form early psychological interventions and subsequent, posttraumatic mental health care. Due to the constant increase in mental health care costs, also post-disaster public mental health requires best possible, cost-effective care systems. Screening and monitoring the affected population might be one such area to optimize the charges.

**Methods:**

This paper analyzes the potential cost-effectiveness of monitoring a psychologically traumatized population and to motivate individuals at risk to seek early treatment. As basis for our model served Grossman's health production function, which was modified according to fundamental concepts of cost-benefit analyzes, to match the basic conditions of online monitoring strategies. We then introduce some fundamental concepts of cost-benefit analysis.

**Results:**

When performing cost-benefit analyses, policy makers have to consider both direct costs (caused by treatment) and indirect costs (due to non-productivity). Considering both costs sources we find that the use of Internet-based psychometric screening instruments may reduce the duration of future treatment, psychological burden and treatment costs.

**Conclusion:**

The identification of individuals at risk for PTSD following a disaster may help organizations prevent both the human and the economic costs of this disease. Consequently future research on mental health issues should put more emphasis on the importance of monitoring to detect early PTSD and focus the most effective resources within early treatment and morbidity prevention.

## Background

Large-scale disasters present unique challenges to any public health system. After a disaster, not only physical injuries, but also psychological suffering and traumatization, may be widespread. Psychological suffering is associated with high rates of chronicity and consequent disability, generating costly medical expenditure and employment pension claims. Yet, post-disaster psychological research has primarily focused on identifying and alleviating symptoms of acute stress disorder (ASD) and posttraumatic stress disorder (PTSD). Less research has been directed toward estimating the costs of disasters and potential economic benefits of interventions. Indirect evidence suggests that intervention strategies could have a positive influence on the overall costs of a large-scale disaster. One study found that even modest reductions in PTSD symptoms might lead to an increase in employment, even when symptom levels remain severe [1]. Relieving the economic burden of psychological suffering after traumatic stress should, therefore, be a major focus in disaster research and policy development.

Early screening for symptoms of psychological illness following major disasters is a fundamental element of any strategy to moderate economic effects. The manifest gains to early screening are not unfamiliar. In fact, the contribution to preventive medicine of screening for early detection of disease was first discussed in the late 1960s [2] and extensively investigated in the 1970s [3,4]. One report suggested the necessity of screening in the following way: *Screening, the deliberate examination of substantial segments of the population [...] in a search for disease at its earliest stages is a logical extension of the role of preventive medicine *[5]. A goal of public mental health strategies has been to filter individuals with pathology from screening to therapy. Various potentially valuable psychometric screening instruments are available [6-8].

The cost savings associated with screening and early intervention have been widely studied. Investigations have been pursued in diverse fields. A significant body of research has been dedicated to early detection of breast cancer, given its clear relevance to reducing mortality. Mandelblatt *et al*. [9] reviewed cost-effectiveness studies of extending breast cancer screening to women over 65. They estimated that extending biennial breast cancer screening to the age of 75 or 80 would cost between US$34,000 and $88,000 at 2002 values, concluding that *biennial cancer screening after age 65 reduces mortality at a reasonable cost for women without significant comorbidity *[9]. A less costly intervention, though one with profound importance, was applied in the setting of hospital malnutrition. In this study, Kruizenga *et al*. [10] used the Short Nutritional Assessment Questionnaire (SNAQ) to improve recognition of malnutrition at hospital admission from 50% to 80%, which significantly reduced hospitalization length. The cost analysis further found that, on average, a mere US$91 at 2005 value investment was required to reduce a hospital stay by 1 day.

Although recent decades have witnessed important progress in economic research of health care and prevention issues, few available studies have used economic theory to model the economic influence of early detection efforts [11]. The objective of this paper is therefore to construct a model that evaluates the cost effectiveness of online early-intervention screening tools. In this study, we demonstrate, theoretically, that online screening for psychological illness following a major disaster produces economic benefits that outweigh its costs. Our analysis is built upon Grossman's application of human capital theory to investments in health [12].

### Health Costs

Interest in health economics has increased considerably over the last several decades [13], motivated by Arrow's pioneering 1963 work [14], which provided economists with a novel framework for analyzing health and healthcare. Greater availability of data and the growing policy process in health care provision have since stimulated a great deal of research. Indeed, an economic approach to health matters has great policy relevance [15], particularly in light of the exponential recent growth in expenditure. In 2003, health costs in Switzerland amounted to 49.9 billion Swiss francs, or 11.5% of the gross domestic product [16]. In 1985, health's share of GDP was just 7.7% [17]. The U.S., the world's largest consumer of health care, has also experienced intensive growth. Expenditures grew from 5.3% of the GDP in 1960 to 8.9% in 1980, 12.2% in 1990 and 14.1% in 2001 [18]. Unfortunately, early data on the costs of mental health care in Switzerland are limited. There is, however, reason to believe that mental health costs are increasing. In 2004, psychotherapy costs were roughly 500 million Swiss francs [19], while inpatient psychiatric treatment totaled 1.8 billion [20].

### Investment *in Health: Grossman's Health Production Function*

In 1972, Grossman, inspired by Becker's *Human Capital *Theory [21], formulated a model in which expenditure in health care were treated as an investment in human capital, similar to education [12]. Grossman's work implied that a rational individual's investment in current health promotion yields returns to health and longevity in later periods. The individual may optimize his personal lifespan by adjusting for changes in the discount rate [22]. There are some crucial differences from traditional demand theory in Grossman's model, because of the particular form of health-related products. Folland, Goodman, & Stano [18] point out that:

• Consumers are not interested in medical care, but rather in health. They request and consume goods that produce health.

• The purchase of health-related goods from the market is not passive. Consumers produce health by investing time in activities that maintain or improve their health status.

• Health, like capital goods, does not depreciate immediately.

• Health is both a consumption and investment good, and investing in health capital leads to future savings. For example, investing in health-promoting activities today may result in greater number of available days of non-illness.

This final point is fundamental to our analysis, because it introduces the concept of investment in health as a way of realizing future economic savings from better mental health status or, correspondingly, to fewer illness days. Hence, the consultation of an online screening tool is interpreted as an investment in human capital.

## Results of Cost-Benefit Analysis

### Investment

In a cost-benefit analysis, an individual compares an investment outlay today with the present value of an expected future return from the investment. The investment outlay amounts to the total costs of engaging in the activity that is assumed to subsequently generate a return. The present value (PV) results from:

$$PV=\frac{FutureAmount}{{\left(1+DiscountRate\right)}^{t}},$$

where the denominator discounts the future return in period *t *to account the time value of money. The cost-benefit comparison is achieved by subtracting the amount currently invested from the calculated present value of future returns. The result is the net present value (i.e. *NPV = PV – current investment*). If the net present value is greater than zero, then the investment should be undertaken.

### Costs

The investment in online psychological screening includes two basic types of costs: direct and indirect [23]. In our model, two types of direct costs are considered. Initial direct costs (IDC) refer to time costs associated with self-administration of the evaluation. Therapy costs represent the monetary value of mental health intervention and treatment for individuals who seek counseling after assessment. Indirect costs in our model encompass employment losses (e.g., lost work days and decreased productivity) and foregone earnings [24].

### Expected Benefits

Economic benefits of an online screening tool will accrue via early detection of psychological disorder. Early detection increases the likelihood of prompt recovery from mental illness, and decreases its rate of chronicity and disablement in the long term. It also reduces the duration of treatment, due to prompt entry into therapy, when mental illness is still at an early stage. The expected benefits may thus be seen as the financial savings that occur from shortening the duration of costly psychotherapeutic care and moderating adverse labor market effects.

## The Model

### Model Structure with 1 Person

Consider a person A, who has experienced a traumatic event such as the Indian Ocean Tsunami Earthquake on December 26, 2004, and develops psychological symptoms associated with the experience. An online self-evaluation test is available, and the individual can choose whether to take the evaluation or not. We assume the following scenarios:

***Scenario 1 ***– *Person A takes the test and has the following cost structure:*

*IDC*_0_(*A*_1_) > 0: Initial Direct Costs of person A in period *t *= 0

*STC*_1_(*A*_1_) > 0: Shot-term Therapy Costs of person A in period *t *= 1

***Scenario 2 ***– *Person A does not take the test and has the following cost structure:*

*IDC*_0_(*A*_2_) = 0

*LTC*_1_(*A*_2_) > 0: Long-term Therapy Costs of person A in period *t *= 1

We further assume that:

(i) There are only two periods. In period *t *= 0, it is possible to take the test and in *t *= 1, therapy occurs.

(ii) In order to simplify the model, we chose a one-period therapy. The therapy begun at an early stage of psychological disease is shorter in duration than one entered at a later stage. Consequently, *STC*_1_(*A*_1_) <*LTC*_1_(*A*_2_).

(iii) The indirect costs of performing the test can only be greater than zero if we consider economic opportunity costs (e.g., a loss of productivity at work).

(iv) Individuals make their decisions with perfect information. This assumption implies that every individual knows about the existence of an online screening test and that individuals have equal access to the online screening test.

The scenarios may now be compared. First, we deduce the present value (*PV*) of costs under the two scenarios. The value of costs under *Scenario 1, PV(S*_1_*) *includes both initial direct costs, at time 0, associated with Person A's participation in the screening assessment, and the discounted value of short-term therapy costs. The value of costs under *Scenario 2, PV(S*_2_*)*, includes only the discounted appraisal of long-term therapy, as Person A does not complete the online tool under *Scenario 2*.

*Present Value under Scenario 1:*

$$PV(S_{1})=IDC_{0}(A_{1})+\frac{STC_{1}(A_{1})}{1+i}$$

*Present Value under Scenario 2:*

$$PV(S_{2})=\frac{LTC_{1}(A_{2})}{1+i}$$

Investments in early detection methods, such as online screening tests, are cost effective as long as the total costs (i.e., short-term therapy costs plus the economic value of initial costs of self-evaluation) are less than the costs that would result from therapy begun at a later stage. Thus, if P*V*(S_1_) <*PV*(S_2_), then by substituting, we arrive at

$$\frac{STC_{1}(A_{1})}{1+i}+IDC_{0}(A_{1})<\frac{LTC_{1}(A_{2})}{1+i}.$$

Rearranging terms yields the following comparison:

(1) $$\frac{LTC_{1}(A_{2})-STC_{1}(A_{1})}{1+i}>IDC_{0}(A_{1})$$

According to equation [1], if the cost reduction resulting from the difference between the therapy costs (left-hand side of the equation) is greater than the direct costs generated by early detection (right-hand side), then the investment in the online screening test should be made.

The next step is to build in indirect costs to the model. A person affected by mental illness after a disaster, such as depression or PTSD, is assumed to be either unable to work or to function at an impaired level productivity. To represent this condition, we add the discounted value of lost earnings to the therapy costs previously estimated, denoting foregone wages with *W*_1_(*A*_*t*_), where *i *= 1, 2.

*Present Value with Indirect Costs under Scenario 1:*

(2) $$IDC_{0}(A_{1})+\frac{STC_{1}(A_{1})+W_{1}(A_{1})}{1+i}$$

*Present Value with Indirect Costs under Scenario 2:*

(3) $$\frac{LTC_{1}(A_{2})+W_{1}(A_{2})}{1+i}$$

If we assume that wage losses for persons who enter therapy later are greater than wage losses for those who begin therapy earlier, after pursuing self-evaluation with the online assessment (i.e., *W*_1_(*A*_2_) > *W*_1_(*A*_1_)), then by differencing equations [2] and [3], subject to the inequality suggested by equation [1], we get the following:

$$\frac{\left[LTC_{1}(A_{2})+W_{1}(A_{2})\right]-\left[STC_{1}(A_{1})+W_{1}(A_{1})\right]}{1+i}>IDC_{0}(A_{1})$$

Rearrange terms gives us

(4) $$\frac{\left[LTC_{1}(A_{2})-STC_{1}(A_{1})\right]+\left[W_{1}(A_{2})-W_{1}(A_{1})\right]}{1+i}>IDC_{0}(A_{1})$$

Once more, as both numerator terms in square brackets are positive, this suggests that early mental illness detection systems are cost saving.

### Model Structure with n Individuals

The aggregate model can now be constructed. This model differs from the 1-person model in that it also accounts for those people who take the screening assessment, but do not develop posttraumatic disorders or a need for therapy. Consequently, in period *t *= 1, the proportion *π *individuals will initiate psychological treatment, and the proportion (1-*π*) will not.

The following statistics offer some insight into the potential range of *π*. Immediately after a disaster, 54% to 59% of all individuals affected develop psychological disorders [25-27]. These numbers decrease to 41% by week 10 and to 22% one year after the disaster [25,28]. In the medium term after a disaster, a variety of disorders may be found, including major depression (20%–41%) [25,29], posttraumatic stress disorder (14%–59%) [25,26,29], different subtypes of anxiety disorder (20%–29%) [25,29] and substance-abuse disorders (14%–52%) [25,30,31]. A potentially traumatic experience (PTE), traced below in Table 1, is a necessary, but not sufficient, cause of chronic PTSD, since pre-traumatic, peri-traumatic, recovery environment and post-trauma lifespan conditions affect the risk of developing posttraumatic difficulties [32]. Therefore, a screening test helps to select and treat those people who suffer from more than a transitory stress response and are predisposed to develop chronic PTSD.

**Table 1:** Cost Path for Scenarios 1 and 2 in the N-Person Model

	**Scenario 1**	**Scenario 2**
*t *= 0	Potentially Traumatic Experience n individuals take the online self-evaluation test. Costs: *n*·*IDC*_0_(*A*_1_)	Potentially Traumatic Experience No one takes the test. Costs = 0
*t *= 1	Proportion *π *need therapy. Costs: *n*·*π*·*STC*_1_(*A*_1_) (1-*π*) are not ill and do not need therapy.	Proportion *π *need therapy. Costs: *n*·*π*·*LTC*_1_(*A*_2_) (1-*π*) are not ill and do not need therapy.
	Resulting total costs: $n\cdot IDC_{0}(A_{1})+\frac{n\cdot \pi \cdot STC_{1}(A_{1})}{\left(1+i\right)}$	Resulting total costs: $\frac{n\cdot \pi \cdot LTC_{1}(A_{2})}{\left(1+i\right)}$

Table 1 illustrates the cost paths of the two previously presented scenarios for the n-person model. In *Scenario 1, n *people (i.e., the whole population who experienced the trauma and was potentially at risk for PTSD) take the available online self-evaluation test in period *t *= 0. Of the *n *individuals, only the proportion *π *requires therapeutic intervention at period *t *= 1. Individuals who do not manifest any mental disease will not need treatment and thus generate no additional costs. The total costs resulting from *Scenario 1 *are reported in the bottom row of the table.

In *Scenario 2*, none of the *n *persons performs the test at time *t *= 0. However, in period *t *= 1, the same number of people, *π*, as in *Scenario 1 *require psychological treatment. The aggregate model continues to maintain that the delayed therapy initiated under *Scenario 2 *carries on longer than under *Scenario 1 *and is therefore more costly. As a result, investments in the test reduce the total costs of mental illness following a traumatic experience.

Beginning with equation [4], this additional assumption can be modeled. We multiply both the left and the right equation by *n*. Since in period *t *= 1, only *π *individuals need therapy, we also multiply the left side of the equation by *π*. We consider *π *as a probability, which takes the value 0 when no one needs therapy and 1 (equal to 100%) when every one needs therapy. Thus, *π *∈ [0; 1].

$$n\cdot \pi \frac{\left[LTC_{1}(A_{2})-STC_{1}(A_{1})\right]+\left[W_{1}(A_{2})-W_{1}(A_{1})\right]}{1+i}>n\cdot IDC_{0}(A_{1})$$

Dividing both sides by *n*·*π *yields

(5) $$\frac{\left[LTC_{1}(A_{2})-STC_{1}(A_{1})\right]+\left[W_{1}(A_{2})-W_{1}(A_{1})\right]}{1+i}>\frac{IDC_{0}(A_{1})}{\pi }$$

Equation [5] differs from equation [4] in one important aspect, seen in the term on the right-hand side of the inequality. In the aggregate state, the initial investment costs must be adjusted for the probability of therapy, *π*, to be compared to the discounted value of total future expenditures. Given that *π *cannot be greater than 1, the resulting adjusted initial direct costs *IDC*_0 _in the aggregate state will be greater than or equal to the investment costs without the adjustment for probability of therapy. Analogous to the former result, investment in early detection methods is cost saving as long as the inequality holds.

## Discussion

There are limitations to our model. One possible shortcoming is that, due to ease of access to the test, many of the individuals who are screened will not be psychologically ill, and will therefore generate unnecessary investment costs. Nevertheless, it could be argued that healthy people have little reason to participate in a screening test for mental diseases, and are unlikely to devote the effort necessary to locate such instruments on the Internet. We also assume that psychologically healthy people are averse to the risk of being observed taking a psychological self-evaluation at work, in order to avoid suspicions about their actual psychological health. For the same reason, one might assume that people with very mild PTSD or other mental health diseases would take the test privately. Empirical results show that individuals with chronic conditions are more likely to use the Internet in order to search information on health issues than those without chronic conditions [33].

A further critique is that the theory that underlies the basis of this model assumes a constant depreciation of human capital stock over time, whereas we must realistically suppose that trajectories of well being will not decline uniformly, but rather will vary according to the presence of health insults. During illness, health stock depreciates faster than during periods of non-illness. This may be so even when individuals make investments in health-improving products and services. Although this criticism does not change the results of the model presented, it nonetheless suggests that the gains to mental health from investing in an online screening tool could differ by the extent of the posttraumatic effect. Assuming consistent therapeutic quality across individuals, these differences will imply varying patterns of cost savings.

Concerning the model itself, there are two points where criticism might be raised. The first is the assumption that the test is 100% reliable, which is unlikely. At this very early stage of research in that particular field, it is hard to estimate how reliable these tests can be. This precursory model aims to show the potential savings offered by an accurate screening, which, even though not reaching an efficiency of 100%, offers significant opportunities to reduce both psychological burden and treatment (social) costs. Secondly, it may be argued that the model does not take into account the natural resolution of symptoms without intervention. Thus the term *π *should be smaller in the second scenario. From a mathematical point of view it has been assumed that the number of individuals that need a therapy is given and remains constant over time, since over time natural resolution of symptoms in some individuals may be offset by aggravate condition in others resulting in a neutral net impact on costs.

## Conclusion

The main objective of this study was to model the investment in online tools for early detection of mental illness and to show its cost savings. Increasing mental health costs are a crucial issue within the context of public health policy. Thus, techniques for bringing down the burdensome costs of diffuse diseases are more necessary than ever. Investment in early detection screening methods can yield great future reductions in costs and duration of illness, providing a beneficial effect on both population health and labor-market productivity due to reduced periods of disablement.

## Competing interests

The author(s) declare that they have no competing interests.

## Authors' contributions

FdG has given substantial contributions to conception, main analysis, hypothesis building, and writing of the manuscript. He has given final approval of the version to be published. WTG participated in the hypothesis building and revised the manuscript critically. JIB was involved in writing and revising the manuscript critically. AR performed the statistical analyses, was involved in writing and has given final approval of the version to be published. JE and SV have revised the manuscript critically. All authors read and an approved the final manuscript.
